# Nanoparticles for Diagnosis and Target Therapy in Pediatric Brain Cancers

**DOI:** 10.3390/diagnostics12010173

**Published:** 2022-01-12

**Authors:** Clara Guido, Clara Baldari, Gabriele Maiorano, Angela Mastronuzzi, Andrea Carai, Concetta Quintarelli, Biagio De Angelis, Barbara Cortese, Giuseppe Gigli, Ilaria Elena Palamà

**Affiliations:** 1 Department of Mathematics and Physics, University of Salento, Monteroni Street, 73100 Lecce, Italy; clara.guido@unisalento.it (C.G.); clara.baldari@unisalento.it (C.B.); giuseppe.gigli@unisalento.it (G.G.); 2 Nanotechnology Institute, CNR-NANOTEC, Monteroni Street, 73100 Lecce, Italy; gabriele.maiorano@nanotec.cnr.it; 3 Neuro-Oncology Unit, Department of Onco-Haematology, Cell Therapy, Gene Therapy and Haemopoietic Transplant, IRCCS Bambino Gesù Children’s Hospital, 00165 Rome, Italy; angela.mastronuzzi@opbg.net; 4 Neurosurgery Unit, Department of Neurosciences, IRCCS Bambino Gesù Children’s Hospital, 00165 Rome, Italy; andrea.carai@opbg.net; 5 Department Onco-Haematology, and Cell and Gene Therapy, IRCCS Bambino Gesù Children’s Hospital, 00165 Rome, Italy; concetta.quintarelli@opbg.net (C.Q.); biagio.deangelis@opbg.net (B.D.A.); 6 Department of Clinical Medicine and Surgery, University of Naples Federico II, 80138 Naples, Italy; 7 Nanotechnology Institute, CNR-NANOTEC, c/o La Sapienza University, Piazzale A. Moro, 00165 Rome, Italy; barbara.cortese@nanotec.cnr.it

**Keywords:** pediatric brain cancers, blood–brain barrier, nanocarriers

## Abstract

Pediatric brain tumors represent the most common types of childhood cancer and novel diagnostic and therapeutic solutions are urgently needed. The gold standard treatment option for brain cancers in children, as in adults, is tumor resection followed by radio- and chemotherapy, but with discouraging therapeutic results. In particular, the last two treatments are often associated to significant neurotoxicity in the developing brain of a child, with resulting disabilities such as cognitive problems, neuroendocrine, and neurosensory dysfunctions/deficits. Nanoparticles have been increasingly and thoroughly investigated as they show great promises as diagnostic tools and vectors for gene/drug therapy for pediatric brain cancer due to their ability to cross the blood–brain barrier. In this review we will discuss the developments of nanoparticle-based strategies as novel precision nanomedicine tools for diagnosis and therapy in pediatric brain cancers, with a particular focus on targeting strategies to overcome the main physiological obstacles that are represented by blood–brain barrier.

## 1. Introduction

Central nervous system (CNS) tumors represent the primary cause of cancer-related death in children [1,2,3]. Treatment modalities include surgery, radiotherapy, and chemotherapy with outcomes largely depending on the biological aggressiveness of the disease and marginal improvement in the last decade [4]. Delivering diagnostic and therapeutic agents to central nervous system represents a critical challenge due to the presence of the blood–brain barrier (BBB) that represents a dynamic, semi-permeable barrier whose function is to protect nervous system microenvironment from pathogens or toxins, separating blood from brain [5]. The tight junctions between endothelial cells along with the basal membrane, characterized by laminin, collagen, and fibronectin, in which contractile pericytes are located, limit the passage or transport of solutes. Additionally, in the BBB astrocytic end-feet are also involved in blocking the free diffusion of molecules. Moreover, some brain regions as the pons have a more restrictive BBB towards the entry of molecules compared with other brain areas, this may have significant consequences for preclinical drug penetration studies and for the treatment of CNS tumors, particularly in diffuse intrinsic pontine glioma (DIPG) [6]. Moreover, BBB allows the passive diffusion of small size molecules such as the indispensable gas oxygen, water, and some hydrophobic solute, as well as lipophilic molecules with a positive surface charge, the latter representing the majority of current diagnostic and therapeutic agents. Meanwhile, the passage of polar components is only permitted by the presence of specific carriers through an active transport (i.e., glucose and amino acids) [7]. Additional BBB crossing mechanisms such as receptor-mediated transcytosis and adsorptive transcytosis occur, and they are currently being explored in order to expand the repertoire of drugs able to pharmacologically penetrate BBB [8]. For example, the most common modification of water-soluble drugs take place through the addition of lipid for allowing passive diffusion into the brain [9]; also, the development of peptide–drug conjugates with a transportable peptide such as insulin, transferrin, etc., through a disulfide bond. In this way, the resultant chimeric peptide is internalized through receptor-mediated transcytosis and then subjected to the action of disulfide reductases into the brain, thus allowing the cleavage and the release of the conjugated drugs [10]. Another strategy involves to the development of pro-drugs able to cross the BBB, which could be (bio)converted into the active drug once reached CNS. All these efforts highlight the urgent need of alternative solutions in pediatric neuro-oncology, and nanoparticles (NPs) can pave the way to novel therapeutic and diagnostic agents. In particular, the development of innovative NPs for early cancer detection is of paramount importance for the possibility of enlarging the therapeutic window, thus improving the prognosis. Nowadays, the routinely employed radiological imaging technique for diagnosing brain tumors is magnetic resonance imaging (MRI) based on gadolinium (Gd) complexes as a contrast agent. This approach is also exploited for surgical planning and surgical navigation, as well as for the postoperative assessments [11]. The clarity of brain cancer areas in gadolinium-based MRI mainly relies on the accumulation of contrast agents in the target region for its ability to cross the BBB alterations and irregularities in the tumor region [12,13]. However, the extent of BBB defects and openings that are responsible for its increased permeability is not equally distributed to the entire cancer edges in several fast-growing type of CNS tumors [14]. Consequently, gadolinium-based MRI could provide suboptimal tumor images, thus resulting in partial surgical debulking, whereas an extra wide resection could certainly positively affect the prognosis [14,15]. In a comparable manner, low-grade primary brain cancers show a low degree of BBB alteration, thus hindering clear tumor limit delineation that is of fundamental importance for successful surgical treatments [16]. Efficient gadolinium-based scans require a significant amount contrast agent; however, concerns about the toxicity of gadolinium-based complexes resulted in restriction of the use of some linear gadolinium for MRI body scans, and authorizations for others were suspended by the European Medicines Agency (EMA) in 2017, similarly the Food and Drug Administration (FDA) has also limited the use of gadolinium-based contrast agents [17,18]. Therefore, contrast agent based on nanotechnology could offer novel opportunities for early and accurate diagnosis, as well as being at the frontier of non-invasive tumor grading. NPs are characterized for their targeting ability directly to the site of interest with a spatio-temporal control of the payload release, additionally NPs can improve stability, bioavailability, and bioactivity of poorly exploited compounds because of their unfavorable pharmacokinetic profiles. Recently, liposomes, polymeric micelles, dendrimers, polymeric, and inorganic NPs have been proven to be effective for brain tumor treatment due to their ability to cross the BBB [19,20,21,22,23,24,25,26], similarly, engineered solid metal NPs such as iron oxide NPs, quantum dots, dendrimers, and lipid NPs have demonstrated their potential as diagnostic tools for the detection of brain cancers [27]. Despite this enormous potential, several factors have to be carefully evaluated as, for example, cell toxicity, clearance, and BBB flow out. Additionally, some clinical trials are still subject to approval and their application in the pediatric population is largely based on results in adults [28]. Routes of nanocarriers delivery in brain tumors.

**Figure 1:**
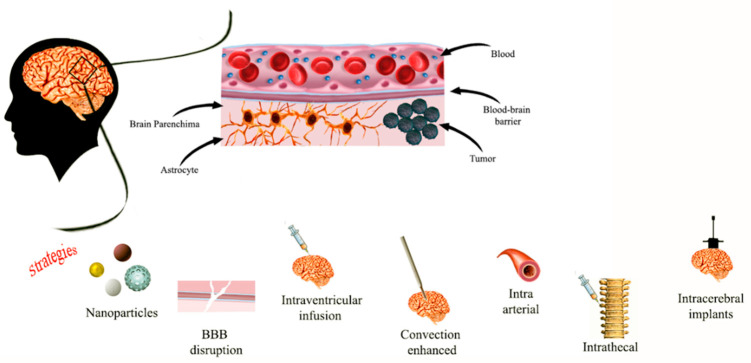
Representative images of different delivery routes to CNS.

Impermeability of the BBB is one well-known mechanism of chemotherapy failure in patients with CNS tumors, leading to an urgent clinical need for alternative strategies [29]. One of these approaches involves the intracerebroventricular and intrathecal administration, which allows a much lower dose of therapeutics when compared with systemic administration. Although this delivery strategy has been applied in pediatric brain cancers [30], it is associated with several limitations, such as the ineffective volume of drug, and it also has limited practicality or effectiveness for the treatment of intraparenchymal tumors. These issues can be addressed by using the convection-enhanced delivery (CED) in which intracranial catheters, stereo-tactically positioned to target sites of interest in the brain, can deliver therapeutic drug by exploiting convection instead of diffusion, over a period of time ranging from a few hours to days (Figure 1).

Clinical or radiological response had been documented in glioblastoma, especially in children who received chemotherapy [31]. CED represents a therapeutic strategy in the brain stem of children with DIPG previously irradiated [32,33] holding the promise of achieving local disease control. Future advances will involve developing therapeutic agents for delivery *via* CED, and improving the technique [32]. In this context, nanotechnology-based delivery systems designed for use with CED could be of aid [34]. Despite this, it turns out to be an invasive and technically challenging solution. An alternative strategy concerns the application of osmotic methods or focused ultrasounds to allow a transient opening of the tight junctions in the BBB. Therapeutic agents can cross BBB, thus achieving good results in the treatment of pediatric brain tumors as primary CNS lymphoma [35]. However, this approach is characterized by several limitations, such as the potential aspecific disruption of the BBB at the tumor area as well as of the adjacent, healthy normal brain. Moreover, once the BBB is altered, not only infused drugs, but also other undesired molecules or toxins responsible for side effects can enter the CNS. Lower toxicities and better survival rates are achieved if nanocarriers are delivered into the bloodstream directly through carotid artery avoiding the systemic circulation, or indirectly towards oral, nasal, and peritoneal routes [36]. Regardless of the administration route, NPs are characterized by a size, small enough to allow an easy elimination from the body, as well as being biodegradable, and having a non-toxic profile. Despite the fact that nanotechnology could represent, in principle, a valid, non-invasive approach to treat pediatric brain tumors, several aspects have to be addressed, such as the lack of animal models, and ethical issues.

In Table 1 are reported different NPs with relative use, advantages, disadvantages, and that are discussed in the review.

In Table 2 are reported clinical trials using NPs in pediatric brain tumors (ClinicalTrial.gov, data are collected by 28 December 2021).

## 2. Inorganic Nano-Systems for Pediatric Brain Cancer Diagnosis and Therapy

### 2.1. Gold Nanoparticles

**Figure 2:**
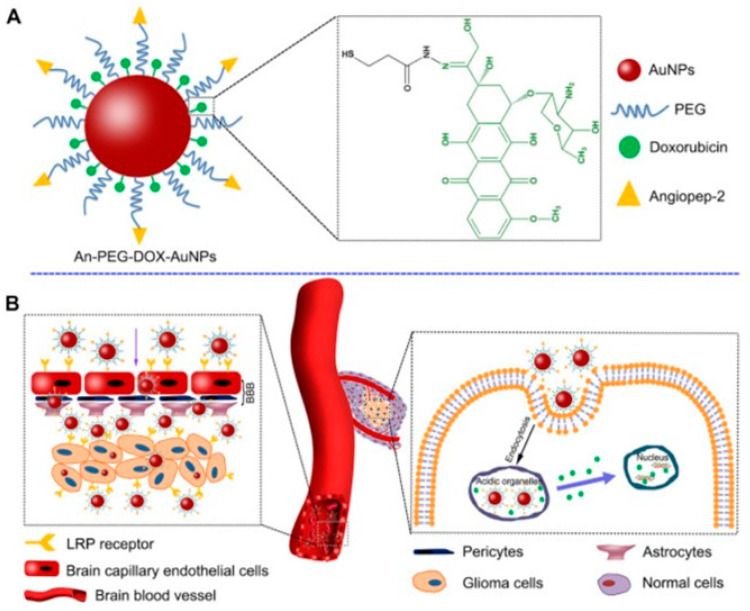
Scheme of the components of engineered AuNP functionalized with drug (green), targeting peptide (yellow) and components (**A**) and the delivery mechanism to glioma (**B**). Reprinted with permission from ref. [38]. Copyright 2014 Elsevier Ltd.

Gold NPs (AuNPs) with a size < 10 nm are used in brain cancer diagnostic and treatments in order to deliver chemotherapeutic drugs, genes, or combined with radiotherapy [22,31]. AuNPs are FDA approved thanks to their small size, good biocompatibility, and the ability to cross BBB without causing damage [22]. Additionally, easy surface modification and controlled drug release make these NPs attractive [35]. AuNPs cross the BBB by passive diffusion (through ion channels such as Ca^2+^, Na^+^, and K^+^ channels), carrier-mediated and receptor-mediated transport (usually based on binding of transferrin modified AuNPs to transferrin receptor highly expressed on BBB epithelium cells) or adsorption mediated endocytosis [22]. Bredlau et al. reports the use of AuNPs conjugated with temozolomide as possible innovative treatment of recurring malignant glioma [42]. Another study [38] reports the efficiency of AuNPs for the delivery of doxorubicin (DOX) against human glioma cell lines, thanks to the employment of Agiopeptide-2 as targeting polymer and polyethylene glycol (PEG) to evade immune recognition (Figure 2).

AuNPs are also exploited as adjuvant for radiotherapy since they increase DNA damage induced by ionizing radiation, as well as the blockage of angiogenesis. Joh et al. employed PEG-functionalized gold NPs coupled to the administration of ionizing radiation in human glioblastoma multiforme (GBM). The combination of these NPs and radiotherapy increased the survival of mice with orthotopic GBM tumors through a specific radio-sensitization [42,69,70]. For the possibility to finely control the physico-chemical properties of AuNPs (i.e., size, shape, and surface roughness), the resulting light absorption ability can be finely tuned, thus making AuNPs attractive for photothermal cancer therapy [37,71,72,73]. As, for example, photothermal therapy based on AuNPs-conjugated to RGD (arginine–glycine–aspartate) peptide were employed to target the integrins that are overexpressed in glioma cells in order to enhance the targeting [42,74]. In a similar manner, AuNPs functionalized with epidermal growth factor or transferrin receptors were loaded with the photosensitizer silicon phthalocyanine (Pc 4) and then employed in photodynamic therapy of glioblastoma. Through this strategy, cytotoxicity was reduced, and the efficacy of drug delivery was ameliorated [42,75]. In another study, AuNPs have been used to deliver short interfering RNA (siRNA) capable of crossing the BBB and to induce apoptosis; favorable outcomes were obtained, in particular a reduction in the tumor progression in xenografts models of GBM without side effects was clearly assessed. The biological target of this siRNA was the oncoprotein Bcl2Like12 (Bcl2L12), an effector caspase and p53 inhibitor overexpressed in GBM. This treatment caused tumor cells apoptosis by enhancing effector caspase and p53 activity [39,42]. Liu et al. have developed AuNPs coated by PEG, chitosan, and polyethyleneimine that were loaded with apurinic endonuclease 1 (Ape 1) siRNA for their successful delivery to pediatric ependymoma (EP) and medulloblastoma (MB) cells [40]. In another study, AuNPs were loaded with DOX and functionalized with PEG and the transactivator of transcription (TAT) in order to enhance the interaction between AuNPs and endothelial cell membranes. These NPs have shown accumulation in glioma cells and enhanced cytotoxicity in vitro and in vivo, when compared to DOX alone [22,41]. Different researches have also highlighted the potential of AuNPs as multifunctional tools for simultaneous brain cancer imaging and therapy. In particular, AuNPs could be used as diagnostic tool thanks their accumulation in the cancer brain tissue and, after irradiation, as therapeutic tools [69,76].

### 2.2. Silver Nanoparticles

Silver NPs (AgNPs) are colloidal metallic NPs widely employed in biomedicine [22,42]. The antibacterial and anticancer effects of AgNPs are mainly due to the release of Ag ions in tumor cells, thus leading to reactive oxygen species (ROS) generation and a subsequent oxidative damage to biological molecules that results in cell death [46,77]. More in details, AgNPs have a toxic effect on mitochondria with disruption of the respiratory chain, excessive oxidative stress, and inhibition of ATP synthesis, factors involved in the activation of apoptotic pathway. Furthermore, AgNPs with a diameter < 10 nm can cross the nuclear pores leading to ROS production and then, to cell cycle arrest and chromosomal aberration in glioblastoma cells [78], thus making attractive their application for increasing GBM cells apoptosis in vitro. In addition, AgNPs were used as sensitizers for radiotherapy [42]. Salazar-García et al. demonstrated the toxic effect of AgNPs and ZnCl_2_ in C6 rat glioma cells [44], showing a decrease in mitochondrial activity (about 13 and 21%, respectively) when used as single agents; on the other hand, combinatorial treatment with AgNPs and ZnCl_2_ decreased more significantly cell viability (around 30% administrating ZnCl_2_ as pre-treatment and 90% in concomitant administration) and led to an efficient apoptosis of C6 rat glioma cells [44].

### 2.3. Iron and Zinc Oxide NPs

**Figure 3:**
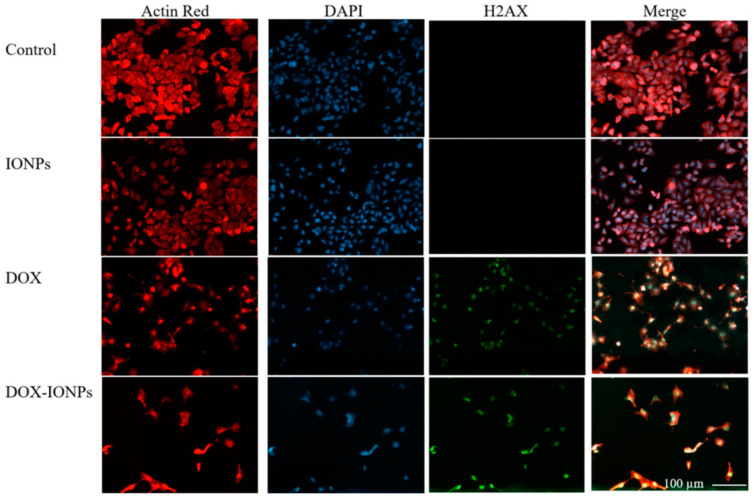
Fluorescent images of glioblastoma U251 cells after treatment with different formulations such as IONPs, DOX, and DOX loaded IONPs. Reprinted with permission from ref. [45]. Copyright 2020 Norouzi, M.; Yathindranath, V.; Thliveris, J.A.; Kopec, B.M.; Siahaan, T.J.; Miller, D.W.

Iron oxide (Fe_3_O_4_) NPs have extensive applications in cancer therapy as drug delivery agent or as magnetic guidance [42,45]. Fe_3_O_4_ NPs are biocompatible and biodegradable, and can be further functionalized in order to enhance biocompatibility, aqueous solubility, and to prolong their circulation time. Norouzi et al. [45], delivered DOX with bio magnetic Fe_3_O_4_ NPs (IONPs) for glioblastoma treatments (Figure 3).

Kievit et al. developed Fe_3_O_4_ NPs with a polymeric shell of chitosan, PEG, and polyethylenimine PEI that binded, protected, and delivered Ape 1 siRNA to perinuclear region of EP and MB cells, thus showing about 75% of down expression in pediatric tumor cells after radiation [79]. Duong et al. developed superparamagnetic Fe_3_O_4_ NPs (SPIONs) loaded with siRNA against MXD3 and observed cell apoptosis in neuroblastoma cell lines. In addition, Fe_3_O_4_ NPs loaded with MXD3 siRNA were combined with DOX, vincristine, cisplatin, or maphosphamide. Results showed an additive efficacy against neuroblastoma [49]. Iron oxide NPs have several advantages such as enhanced targeting, and delivering to diseased tissues *via* a magnetic field. Furthermore, once having been taken up by tumor cells, these NPs can be activated externally by alternating a magnetic field to destroy surrounding the target tissue through hyperthermia [80]. In this frame, SPION were coupled with an anti-EGFR antibody, which was expressed in human glioblastoma multiforme (GBM), and then used for MRI directed CED in cancer therapy [50]. Additionally, SPIONs represent a promising T2 MRI contrast agent that can persist for long time in the brain tissue, thus precisely defining the cancer edges with respect to gadolinium-based T1 MRI contrast agents [81,82]. Reddy et al. [51], developed a multifunctional SPION able to target glioma cells and to provide diagnostic images by using MRI and fluorescence. Their results showed the capability of this multifunctional SPION to penetrate the BBB and to mark brain cancer cells.

Another class of inorganic NPs is represented by zinc oxide (ZnO) NPs that are biocompatible and can be used for biomedical application [48,83]. The oral delivery of engineered ZnO NPs allows the brain to be reached and the BBB to be breached [46]. Wahab et al., showed that ZnO NPs present anticancer properties in the glioblastoma cell line [47]. The strong anticancer effect is due to induction of intracellular ROS generation and activation of apoptotic signaling pathway. Nonetheless, additional studies on ZnO NPs are required, including comparative analysis with other inorganic NPs, to better understand mechanism of potential toxicity in complex biological systems [48].

## 3. Lipid Based Nanoparticles in Pediatric Brain Tumors Treatment

**Figure 4:**
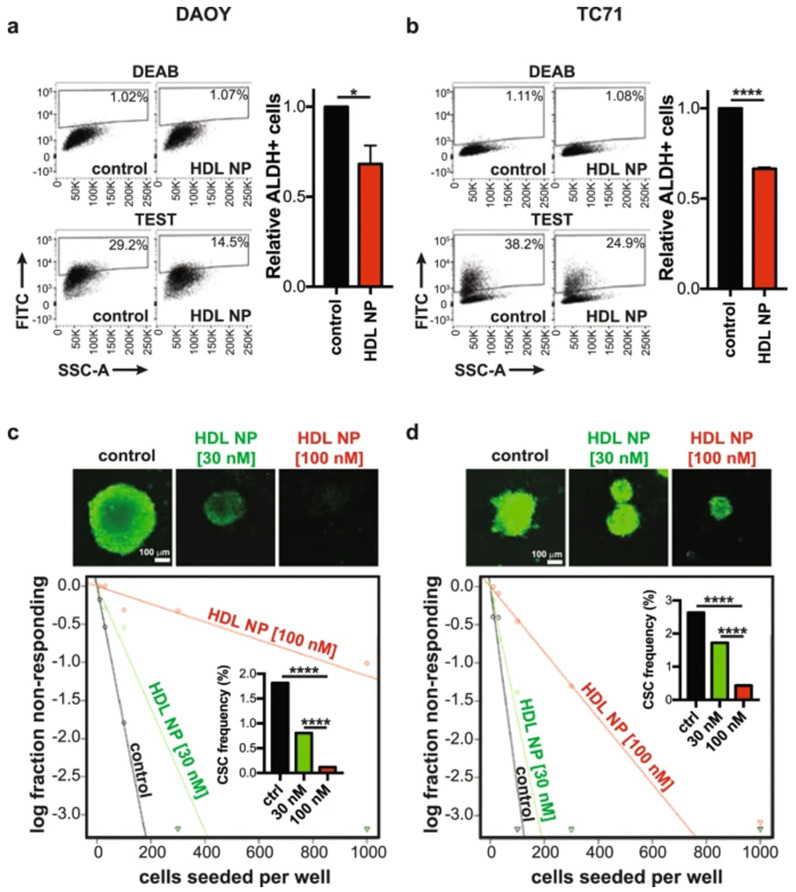
Flow-cytometry analysis of cancer stem cells reduction after treating DAOY cell line: (**a**) and TC71 cell line (**b**) with HDL NPs for 48 h. Spheroids obtained from DAOY (**c**) or TC71 (**d**) cells after dissociation were cultured with or without HDL NPs, in order to evaluate the formation of spheroids. Data are expressed as means ± SEM of 4 independent tests. * *p* ≤ 0.05, **** *p* ≤ 0.0001, and refer to unpaired, two-tailed t-test. Reprinted with permission from ref. [89]. Copyright 2018 Bell, J.B.; Rink, J.S.; Eckerdt, F.; Clymer, J.; Goldman, S.; Thaxton, C.S.; Platanias, L.C.

Liposomes and micelles are organic NPs based on lipids that present different features: (i.) micelles are unilamellar NPs, smaller than liposomes, and are able to load hydrophobic therapeutics inside their structure or bound to their surface; (ii.) liposomes are composed by one or multiple lipid bilayers that form an internal aqueous compartment and a lipophilic external shell. Recently, liposomes have been exploited as drug delivery system against GBM [84], showing a good profile of biocompatibility and biodegradability, low toxicity, and the ability to load both hydrophilic and hydrophobic drugs [22,42,85]. There is evidence that highlights the ability of lipid NPs to accumulate in tumor tissue, due to the enhanced permeability and retention (EPR) effect [86]. The positively charged lipid-derived NPs are able to cross the BBB through adsorption-mediated transcytosis or endocytosis; liposomes conjugated with a specific BBB ligand, such as transferrin, insulin, or endothelial growth factor, can easily target the CNS by endocytosis mediated by a receptor [22,42,87]. Undoubtedly, one of the main drawbacks in the application of lipid NPs for pediatric brain cancers is represented by their rapid clearance. It has been demonstrated that liposomes conjugated with PEG evade opsonin recognition and the following clearance by the reticuloendothelial system (RES), thus prolonging their circulating time [42,46,88]. Many chemotherapeutic agents loaded into liposomes are involved in preclinical studies to treat brain cancers such as GBM, with respect to free drugs. These nanoformulations can accumulate in the tumor site, thus providing improved drug efficacy and lower toxicity [42,46]. Infante et al. developed amphiphilic self-assembling micelles to deliver Glabrescione B (Gla B), a hedgehog (Hh) inhibitor, which drastically inhibited tumor growth in both allograft and orthotopic models of Hh-dependent MB. It was demonstrated that micelles enhanced bioavailability of the poor water solubility of Gla B without the employment of organic solvents, thus obtaining favorable pharmacokinetics with negligible toxicity [52]. Bell et al. developed high density lipoprotein (HDL) NPs targeting the scavenger receptor class B type 1 (SCARB 1). Their results showed antineoplastic effects against the sonic hedgehog subtype of medulloblastoma (SHH-MB) (Figure 4), and potent inhibitor effects on the population of cancer stem cells, which is a result of the paramount importance in preventing tumor recurrence and therapeutic resistance [89].

Additionally, Kim et al. developed biomimetic HDL NPs able to cross the BBB and deliver a SHH inhibitor (LDE 225) to cancer stem cell population of (SHH--MB) in vitro, ex vivo, and in vivo. These NPs were fabricated by incorporating with anti CD15 and apolipoprotein A1 to achieve a dual targeting delivery through binding of SCARB1 and CD15, both expressed by SHH MB cells. These NPs act as drug carriers and show a therapeutic effect due to efficient delivery of LDE 225, and for their intrinsic antineoplastic effect through SCARB1 intracellular cholesterol depletion in SHH MB cells [54,90]. Lipid-based NPs show many advantages like formulation simplicity, biocompatibility, and ability to load both lipophilic and hydrophilic drugs. However, despite these advantages, lipid NPs also show high uptake to the liver and spleen [53], which can induce a strong immune response. This limitation can be addressed via PEGylation of the nanoparticles [91] and, through modifications with cholesterol, it is possible to improve their stability and intracellular trafficking, and to reduce destabilization in the presence of serum [92,93].

Lipid-nanoparticles loaded with contrast agent can also represent an optimal strategy for the diagnosis of brain cancers thanks to their ability to promote the BBB passage of contrast complexes. Yang et al. [94] reported the synthesis of ultrasensitive magnetic resonance contrast agents for the imaging of brain cancers composed by a magnetic nanocrystals core and a coating of di-block of mPEG and dodecanoic acid. MRI investigation showed that these hybrid nanoparticles present good sensitivity against cancer cells with low toxicity in health cells.

## 4. Therapeutic Carbon Dots in Pediatric Glioma Diagnosis and Treatment

Carbon nitride dots (CNDs) are small biocompatible, non-toxic nanoparticles with the ability to selectively target cancer cells [95]. In addition, CNDs are fluorescent nanotools considered as an optimal candidate for cancer diagnosis [96]. Different researchers exploited properly engineered CNDs in combination with other contrast agents, such as gadolinium [97] or Fe^3+^ [98], to improve their cyto-biocompatibility, to inhibit leakage or, in the case of conjugation with Fe^3+^, to improve discrimination between normal and cancerous cells [99].

**Figure 5:**
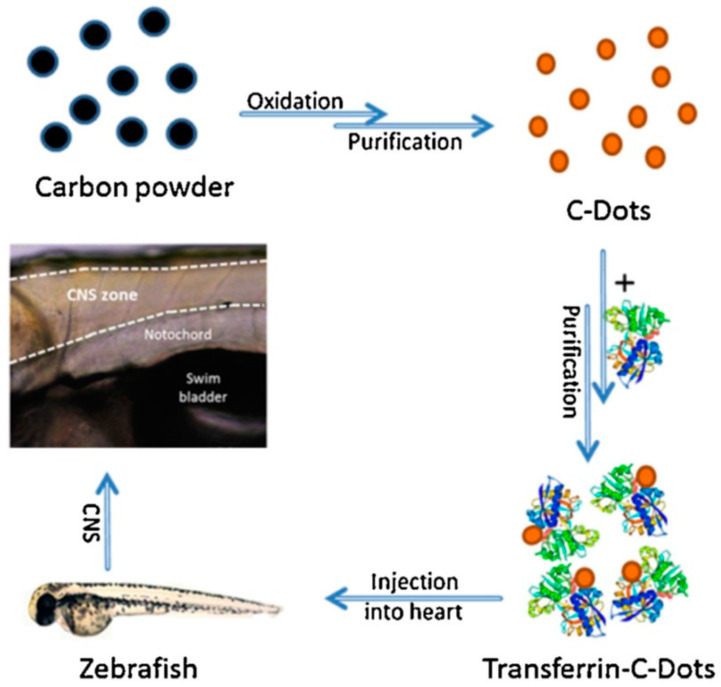
Scheme of fabrication for CNDs functionalized with Tf. After injection in zebrafish, CNDs were able to cross the BBB and to accumulate in the CNS. Reprinted with permission from ref. [58]. Copyright 2016 Elsevier B.V.

Recently, CNDs were used to target pediatric glioma cells by delivering gemcitabine (GM), a drug with anti-glioma effects thanks to the induction of cell death triggered by DNA polymerase inhibition. Despite the potential of GM, its effectiveness often is compromised due to short blood half-life, toxicity, and difficulty in crossing the BBB [56]. CNDs conjugated with GM have been successfully tested not only in SJGBM2 (a high-grade pediatric glioma) cell line but also in normal human embryonic kidney cells [57]. However, CNDs functionalized with transferrin (Tf) can improve the delivery of drugs that have showed poor capacity to penetrate CNS. For example, CNDs functionalized with Tf were employed to study the BBB cross in a in vivo model, such as zebrafish (Figure 5). It was observed that Tf-CNDs easily crossed the BBB with respect to non-functionalized CNDs [58]. Regardless of Tf conjugation, a self-ability of CNDs to cross the BBB could be by mimicking the glutamine structure, whose transport is allowed through the ASCT2 carrier expressed onto endothelial brain cells, in addition to their small size, which allows passage through passive diffusion [56]. Another application of carbon dots for the pediatric brain cancers concerns the delivery of DOX by covalently binding to CNDs, a noticeable fivefold increase in DOX inside the nuclei of cells with a consequent significative decrease in cytotoxic side-effects. However, an effective release of DOX from these nanostructures has not been demonstrated, leading to the hypothesis that the covalent bond between the drug and nanoparticle is quite strong [100].

## 5. Polymeric NPs as Diagnostic Tools and Therapeutic Nanocarriers in Pediatric Brain Malignancies

Polymeric NPs can satisfy several requirements, which allow clinical applications for drug delivery or gene therapy in cancer to be reached [77,101,102], as well as for delivering diagnostic probes [103]. Optical imaging tools were obtained by engineering polymeric fluorescent NPs in order to detect cancer margins. In particular, near infrared (NIR) fluorescent dyes were conjugated with polymeric NPs, and were used in the early tumor discovery in the monitoring of the therapy and in the image-guided surgical treatment [104,105]. The appropriate choice of polymeric building blocks can enhance the pharmacokinetic and pharmacodynamic characteristics of the encapsulated drug, and can offer the controlled release of the drug, which can be triggered by different external stimuli such as pH or temperature, as well as by diffusion-control or solvent-activation [59]. Recently, poly(β-amino ester) (PBAE) NPs were developed for MB and atypical teratoid/rhabdoid tumors (AT/RT), which are among the most common pediatric brain tumors [3]. MB belongs to the most aggressive pediatric brain tumors due to its ability to metastasize in different regions of brain and spinal cord. Chemotherapeutic agents are often used during MB treatment, often combined with irradiation, but the success of this therapy is limited to only a 60% cure rate. Regarding AT/RT, its 10-year survival rate is unacceptable (only 26.2%); even after applying radiotherapy, its survival rate is only extended by just one year. So, considering the need for efficient therapeutic strategies, PBAE NPs, loaded with plasmid DNA encoding the suicide gene of the *Herpes simplex* virus I thymidine kinase (HSVtk), have been developed to treat pediatric MB and AT/RT [62]. The choice of the PBAE polymer is driven by its safety as well as its fast biodegradability, which ensure good efficacy and no toxicity [61]. Achieving more than 50% of effective transfection in both the D425 MB and BT-12 AT/RT cell line, PBAE NPs have shown their killing capabilities after ganciclovir (GCV) treatment in cells expressing HSVtk, thus resulting in more than 65% of death. Nevertheless, in vivo applications have shown a slight extension of survival, from 35 days for untreated mice bearing BT-12 tumors, to 42 days after treatment with HSVtk NPs. In the same way, the survival for mice with MB was extended from 19 to 31 days. PBAE NPs have also found application in gene silencing therapy, achieving an effective delivery of siRNA in tumors derived from human GBM implanted intracranially in mice. Furthermore, the ability of PBAE NPs loaded with two different miRNAs (miR-148a and miR-296-5p) to inhibit the growth of brain cancer cells has been proven, allowing for prolonged survival in mice implanted with human GBM, [63]. Recently, the constant need to find a non-toxic treatment for MB has led to the development of new poly(ethylene glycol)-poly(2-methyl-2-benzoxycarbnyl-propylene carbonate) (PEG-PBC) nanoformulations loaded with the potent BET bromodomain inhibitor JQ1, and conjugated with apolipoprotein E to exploit the overexpression of low density lipoproteins (LDL) receptors on the endothelial cells of BBB [106]. These NPs show a small size of 60 nm, they are quickly internalized in CNS by receptor-mediated endocytosis in the BBB, and avoid immunological clearance from reticuloendothelial system thanks to a PEG shell, to achieve receptor-mediated endocytosis [86]. After their systemic administration, an effective inhibition of MB was achieved in comparison with control and non-targeted NPs loaded with JQ1. Polymeric nanoparticles can also be fabricated by using another attractive polymer represented by the biocompatible PLGA. It has found application in the delivery of paclitaxel in a glioma mouse model [64], as well as in the delivery of shikonin in rat brain [65] and rotigotine in mouse brain [66]. Additionally, PLGA is an excellent building block for the fabrication of nanocarriers for targeted gene therapy, several studies of gene silencing by means of PLGA NPs loaded with specific siRNA have shown a durable gene knockdown as well as a slow degradation of their cargo, thus allowing a prolonged release of the transported therapeutic agent over time [21].

## 6. Dendrimers in Diagnosis and Treatment of Brain Cancer in Children

Dendrimers (DDs) are a nanosized class of brunched polymers that are organized in three-dimensional nano-architecture. In the last decades, dendrimers have been studied as tools for diagnostic and for drug/gene delivery in brain tumors [37,46,107]. DDs are characterized by a central core, brunched units attached to the core and terminal functional groups exposed to the surface [67]. Dendrimers have been successful conjugated with gadolinium-based contrast agents and fluorescent dye in order to achieve high resolution dual diagnostic imaging by using magnetic resonance and fluorescence [107]. Shi et al., reported the development of multifunctional DDs conjugated with folic acid and gold NPs, thus showing excellent targeting of cancer cells and simultaneously their imaging [108].

**Figure 6:**
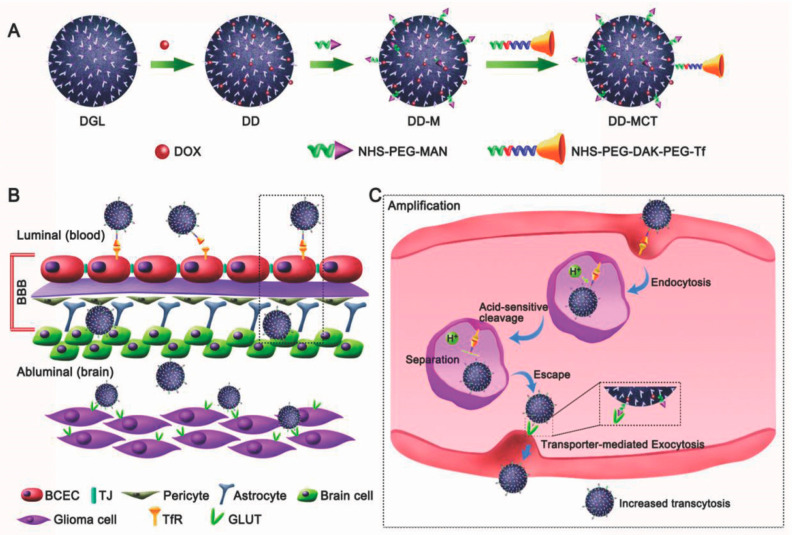
Scheme of the fabrication of acid-sensitive dendrimers (**A**) able to target Tf receptor expressed by BBB and then, GLUT receptor expressed on glioma cells after transcytosis across the BBB (**B**). Schematization of the acid-sensitive cleavage of Tf-dendrimers (**C**). Reprinted with permission from ref. [109]. Copyright 2018 Wiley-VCH Verlag GmbH & Co. KGaA.

DDs present excellent properties such as shape and size, which can be precisely controlled to modulate in vitro and in vivo activities [46,67]. To achieve targeted delivery and selectivity for brain tumor cells (Figure 6), the surface of dendrimers can be covalently decorated with transferrin, thus conferring the ability to cross the BBB through receptor assisted transcytosis [109].

PEGylation and carboxylation are employed to exclude toxic side effects and to evade the undesired immune response [67,110]. In this frame, glycosylation, acetylation, and amino acid functionalization have also been successfully employed to enhance its biocompatibility [46]. The most used DD is based on poly-amidoamine (PAMAM), a water-soluble polymer able to efficiently interact with hydrophobic compounds [46,110]. Liu et al. developed PAMAM dendrimers loaded with DOX and conjugated with a peptide directed to EGFR for targeting glioma cells, and managed to deliver the anticancer compound directly to the target site [29]. In addition, suitable functionalization strategies of PAMAMs resulted in dramatic effects on their ability to diffuse in the CNS tissue and penetrate living neurons as shown in vivo after intraparenchymal or intraventricular injections [30]. DDs are also used as transfection reagent in vitro to deliver plasmid DNA and siRNA for targeted gene therapies to the brain [37]. The advantages of DD for developing diagnostic and therapeutic nanotools include the high control of the architecture, the tunable surface functionality, the possibility to load both hydrophilic and hydrophobic molecules, and the scalability; although, until now, translation in vivo has proven been challenging [52].

## 7. Nanoparticles in Immunotherapy for Pediatric Brain Tumors

Cancer immunotherapies developed for adult brain cancer are not effective for pediatric brain tumors due to the different maturation of immune system; only few clinical trials in pediatric brain tumors are active [111,112,113].

Sayour et al. developed innovative lipid NPs that deliver tumor-derived RNA that quickly activate a T cell response via the MHC presentation, they showed that immunocompetent mice with high-grade glioma responded to treatment with tumor-derived RNA loaded NPs [114]. In addition, Sayour and colleagues developed clinically translatable NPs that can be combined with tumor-derived RNA for near-immediate induction of systemic anti-tumor T-cell immunity against medulloblastoma (MB). They enhanced the immunogenicity by combining different mRNAs encoding for immunomodulatory molecules or by including immune checkpoint inhibitors into the RNA-NPs. The results obtained in a pre-clinical model of cellular immunotherapy targeting an anaplastic murine MB, are promising. Based on these findings, the preclinical safety, efficacy, and immunologic effects of RNA-NPs targeting malignant canine brain tumors are first explored, before human evaluation [115].

Moreover, recently, an immunologic response against pediatric gliomas was explored by the use of RNA cancer vaccines. In this frame, Mendez-Gomez et al. synthesized a multilayer package made of an mRNA backbone for delivering gene transcripts in pediatric brain tumors using a lipid-NPs vehicle. The RNA-NPs localized to myeloid cells in murine KR158b brain tumors and activated dendritic cells that induced an antigen-recall response with long-term survivor benefits. The RNA-NPs reprogrammed the brain tumor microenvironment while inducing a glioma-specific immune response. They received FDA-IND (Food and Drug Administration-Investigational New Drug) approval for first in human trials (IND#BB-19304) in pediatric patients with high-grade gliomas [116].

Lenzen et al. developed small inhibitory (si)RNA oligonucleotides and spherical nucleic acids (SNAs) to therapeutically inhibit the gene expression of immunosuppressive IDO1 (Indoleamine 2, 3-dioxygenase 1) in pediatric diffuse intrinsic pontine glioma. They produced and characterized gold NPs for targeted inhibition of IDO1, which can transverse cellular membranes, are stable in physiological environments, can escape from degradation, and create precise targeting in brain tumors. Custom siRNA targeting IDO1 among exons 9-10 resulted in a significant decrease in overall IDO1 expression in multiple DIPG cell lines [117]. The combination of immunotherapy with nanotechnology will provide novel and promising opportunities to improve brain cancer treatment.

## 8. Conclusions

Solid tumors in children mostly affect SNC and represent a huge therapeutic challenge due to their ability to infiltrate and spread to nearby tissues, thus limiting the application of surgery as possible mono-therapeutic intervention. Despite several advancements in the last years, radiotherapy and chemotherapy also turn out to be ineffective, and are associated with significant and serious side effects such as nephrotoxicity, cardiotoxicity, infertility, and deafness. At the same time, novel diagnostic imaging solutions are urgently required in pediatric neuro-oncology to allow early and accurate cancer detection and, hopefully, even non-invasive tumor grading. Nanotechnology-based solutions hold promise for their ability to cross the BBB and, in combination with the repertoire of available building blocks, fabrication strategies and post-synthesis functionalization approaches may pave the way for advancements in this problematic medical area. However, several issues have to be carefully evaluated, mainly biocompatibility assessment and clearance modulation, as well as a lack of suitable animal models, and ethical issues. Nevertheless, over the last years, different strategies have been adopted in order to overcome these drawbacks, and, along with the growing body of knowledge in the molecular genetics of brain tumors, the scientific community is definitely close to the major breakthrough in the development of efficient, safe, and low-cost nanosystems able to image and treat cancer brain cells without inflicting or sustaining significant damage to healthy tissue.

## Figures and Tables

**Table 1:** Use, advantages, and disadvantages of different NPs.

Nanoparticle	Use	Advantages	Disadvantages	In Vitro/In Vivo Models
Au NPs	− Diagnostic − Drug/gene delivery − Adjuvant for radiotherapy − Photothermal cancer therapy	− Tunable physico-chemical properties − Easy surface modification − Controlled drug release [37] − Ability to cross BBB without causing damage [22]	− Potential cytotoxicity − Unknown long-term biodistribution and immunogenicity [37]	− C6 human glioma cells [38] − Xenografts models of GBM [39] − Ependymoma cells (Res 196) and medulloblastoma cells (UW228-1) [40] − Glioma bearing mice [41]
Ag NPs	Tumor therapy	− Antibacterial properties − Anticancer properties − Sensitizers for radiotherapy [42]	Cytotoxicity in lung, stomach, breast, and endothelial cells [43]	C6 rat glioma cells [44]
Fe_3_O_4_ and ZnO NPs	− Drug/gene delivery agent − Magnetic guidance − Contrast agent − Suitable for hyperthermia (Fe_3_O_4_)	− Biocompatible − Biodegradable − Further functionalization [45] − Oral delivery of ZnO NPs [46] − Anticancer properties (ZnO) [47]	Potential toxicity in complex biological systems (ZnO) [48]	− Glioblastoma U251 cells [45] − Neuroblastoma SH-SY5Y cells [49] − Athymic nude mice [50] − Rat 9L gliomas [51]
Lipid based NPs	− Drug delivery − Diagnosis	− Formulation simplicity − Biocompatibility − Ability to load both lipophilic and hydrophilic drugs [32,52]	− Rapid clearance, increased by PEG conjugation [46] − High uptake to the liver and spleen can be addressed *via* PEGylation [53]	− Allograft and orthotopic models of Hh-dependent MB [52] − SHH MB cells [54]
Carbon dots	− Diagnosis − Treatment	− Easy surface functionalization − Possibility to bind inorganic and organic molecules − Low toxicity [55]	− Short blood half-life − Toxicity − Difficulty in crossing the BBB [56]	− SJGBM2 cell line [57] − Zebrafish [58]
Polymeric NPs	− Drug delivery − Gene therapy − Diagnostic probes delivery	− Stimuli-responsive drug release [59] − Biodegradability, biocompatibility, and non-toxicity [60]	− Potential local immune response [60] − Unknown degradation products [61]	− D425 MB and BT-12 AT/RT cell line [62] − Mice bearing BT-12 [63] − Mice implanted with GBM [63] − Glioma mouse model [64] − Rat brain [65] − Mouse brain [66]
Dendrimers	− Diagnosis − Drug/gene delivery	− High control of the architecture − Easy surface modification − Loading hydrophilic and hydrophobic compounds [67]	Neurotoxicity [68]	− U87-MG cells [29] − Female BALB/c nude mice and female CB-17 SCID mice [29] − Primary mouse cortical cultures [30]

**Table 2:** Clinical trials using NPs in pediatric brain tumors.

Phase	Intervention/Treatment	Recruitment Status	Last Update Posted	Ages Eligible for Study	ClinicalTrials.gov Identifier	Type of Cancer
Phase 1	Irinotecan loaded liposomes	Recruiting	18 September 2019	1 to 20	NCT02013336	NB
Phase 1	Doxorubicin loaded liposomes	Recruiting	25 September 2020	Up to 30 years	NCT02536183	NB
Phase 1	Doxorubicin loaded liposomes	Completed	28 April 2015	Up to 21 years	NCT00019630	BT
Phase 1	Doxorubicin loaded liposomes	Withdrawn	19 March 2019	1 year to 40 years	NCT02557854	NB
Phase 1	Cytarabine loaded liposomes	Unknown	23 March 2010	1 year to 21 years	NCT00003073	CNST
Phase 1	Panobinostat Nanoparticle Formulation MTX110	Completed	15 October 2021	1 year to 17 years	NCT03566199	DIPG
Phase 1	Infusate with MTX110 and gadolinium	Recruiting	8 December 2021	1 year to 17 years	NCT04264143	DIPG, DP, TG, DMD
Not applicable	DSC-MRI with ferumoxytol (small iron particles)	Unknown	1 February 2018	1 year to 17 years	NCT00978562	BN
Phase 2	Combidex (ultra-small iron oxide particle) as MRI contrast agent	Terminated	16 May 2017	1 year to 17 years	NCT00659334	BN

Abbreviations used in the table: Central Nervous Systems tumors (CNST); Brain tumor (BT); Diffuse Intrinsic Pontine Glioma (DPIG); Diffuse Pontine (DP); Thalamic Gliomas (TG); Diffuse Midline Glioma (DMD); Brain Neoplasm (BN).
